# Coded Ultrasonic Ranging for the Distance Measurement of Coaxial Rotor Blades

**DOI:** 10.3390/mi16020240

**Published:** 2025-02-19

**Authors:** Yaohuan Lu, Zhen Qiu, Shan Zhang, Wenchuan Hu, Yongqiang Qiu, Zurong Qiu

**Affiliations:** 1 State Key Laboratory of Precision Measuring Technology and Instruments, Tianjin University, Tianjin 300072, China; luyaohuan@tju.edu.cn (Y.L.); fami_z34@tju.edu.cn (S.Z.); 2 School of Engineering, University of Bolton, Bolton BL3 5AB, UK; z.qiu@bolton.ac.uk; 3 College of Mechanical Engineering, Tianjin University of Technology and Education, Tianjin 300355, China; huwenchuan@tute.edu.cn; 4 School of Engineering, Liverpool John Moores University, Liverpool L3 3AF, UK; y.qiu@ljmu.ac.uk

**Keywords:** blade-tip distance, coded ultrasonic ranging, signal processing, measurement rate

## Abstract

Coaxial rotor helicopters have a wide range of civilian and military applications; however, the collision risk of the upper and lower blades that comes with the coaxial rotor system remains. This paper introduces a blade-tip distance measurement method based on coded ultrasonic ranging to tackle this challenge. Coded ultrasonic ranging with phase modulation was adopted to improve the measurement rate. In this paper, seven-bit M-sequences and Gold codes are chosen with four periods of 200 kHz sine wave carriers as the excitation signals, and the received signals are filtered by a bandpass filter and decoded by a matching filter. The coding performance is evaluated by the distinguishability and energy level of the received signals. The experimental results show that the measurement rate can reach 3060 Hz for a distance of one meter. They also give the potential solution for other high-speed measurement problems.

## 1. Introduction

Compared with traditional single-rotor helicopters, coaxial rotor helicopters have the advantages of a small size, a compact design, high weight efficiency, increased stability, and good maneuverability [1,2,3,4]. However, the dual-rotor system introduces structure complexity and raises the risk of collision between the upper and lower blades. Blade collision usually happens at the blade tips due to the deformation of the blades during the high-speed rotation. In order to prevent this risk, it is necessary to measure the distance between the upper and lower blades in real-time during flight and use the distance information to adjust the flight status. In addition, the method introduced herein can also provide a reference during the design of the coaxial rotor blades.

Fiber-optic sensors are proposed to monitor blade deformation in [5,6]. In their method, a series of fiber-optic sensors are arranged on the surface of the blade, and the strain deflection line of the blade is obtained by fitting and decoupling the strain of the sensors. Due to the discrete distribution of the fiber-optic sensors, the sensor installation angle has a great influence on the measurement accuracy, and the decoupling of strain is relatively difficult. The blade deformation error can reach 5% [6], which causes a larger error in calculating the blade-tip distance from the blade deformation. The visual measurement method is also widely used. In this method, a target or irradiation mark is affixed to the blade surface, and then images of the blade are taken by multiple CCD cameras which are mounted on the ground [7,8,9,10]; subsequently, the mark position and the parameters of the blade tip can be obtained after image processing. However, this method is not suitable for airborne real-time measurement due to the layout requirements for the ground installation of large measuring devices. In addition, the accuracy of visual measurements can be easily influenced by environmental factors. An electromagnetic measurement method is proposed in [11] which entails embedding electric or magnetic field antennas in the upper and lower rotor blades and using the principle of near-field effect. However, this method is still in the theoretical research stage, and actual engineering experiment verification has not been reported yet. Electromagnetic ranging is usually utilized for long distance measurements, at hundreds of meters, but suffers from a low resolution; for example, the resolution is 37.5 mm when using the common 5 GHz electromagnetic bandwidth [12]. In addition, it raises further risk of electromagnetic interference among electronic devices. As a common ranging method, laser measurement has good directivity [13], but blade flapping and lagging and torsion of the blade lead to the laser measurement not being aligned with the measured target, so it is not suitable for the measurement of blade-tip distance.

Ultrasonic ranging is a method of distance measurement based on the time of flight and the speed of ultrasound propagation in the medium. It is suitable for short range measurements within several meters [14], covering the distance between upper and lower rotor blades’ tips (in general 100–1000 mm). For a typical air-coupled ultrasonic transducer of 40 kHz, the wavelength of the propagated wave is 8.5 mm, making the measurement accuracy sufficient for the proposed application. The size of these types of transducers is relatively small, and they can be installed in the blade tip to complete the airborne real-time measurement. In addition, ultrasonic ranging does not cause electromagnetic interference with other electronics on the helicopters.

Ultrasonic ranging can be set up in the pitch-catch mode with a pair of transducers. One transducer is used as a transmitter, and the other one works as a receiver. When applying ultrasonic ranging to real-time blade-tip distance measurement, the challenge arises from the blade moving at a high speed, with rotor speeds generally being between 400 and 500 revolutions per minute (RPM). The intersection of the upper and lower blades happens quickly within a very short time window. On the contrary, the propagation speed of ultrasound is relatively slow, about 340 m/s in the air at 20 °C. If the traditional ultrasonic ranging method is adopted, the transmitter transmits a measurement signal when the upper and lower blades intersect. However, due to the high speed of the blade tips and the relatively slow propagation of ultrasonic waves, the signal cannot travel the full distance and be received by the receiver. By the time the signal travels the full distance, the receiver has moved out of the measurable range of this intersection. Thus, the effective measurement of the blade-tip distance cannot be completed. The time required for a single measurement is limited by the speed of sound, resulting in a relatively low measurement rate which hinders the application of ultrasonic ranging to blade-tip distance measurement.

In this paper, a continuous measurement method based on coded ultrasonic ranging is proposed for measuring blade-tip distance, and it is also potentially suitable for other applications that require high-speed measurement in dynamic scenarios. The traditional method of ultrasonic ranging can only transmit one measurement signal at each intersection, while continuous measurement means that multiple measurement signals can be continuously transmitted at each intersection. Continuous measurement does not need to wait for the previous measurement signal to be received by the receiver before transmitting the next measurement signal. The coded ultrasonic method enables us to add features to the ultrasonic signal, that is, to modulate the amplitude, frequency, phase, pulse position, and so on. Currently, pulse-position modulation [15,16] is the prevailing method for improving the measurement rate of ultrasonic ranging, but the currently achievable measurement rate is not enough for blade-tip distance measurement, and there have been no reports on other coded ranging techniques being utilized. The application of coded ultrasonic ranging to blade-tip distance measurement is first proposed in this paper, so the coded ultrasonic signals are studied in detail, and the suitable coding methods are identified.

This paper is structured as follows: Section 2 introduces the definition of blade-tip distance, the calculation method of blade-tip distance based on coded ultrasonic ranging, and the method of improving the ultrasonic measurement rate; Section 3 presents the measurement system for coded ultrasonic ranging, the specific coding and decoding method, and the performance evaluation method for coded ultrasonic ranging; Section 4 presents the experimental results, including the results of the coding, decoding, and performance evaluation; Section 5 summarizes the advantages and disadvantages of the proposed method.

## 2. Methodology of Blade-Tip Distance Measurement Based on Ultrasonic Ranging

### 2.1. Measurement Method of Blade-Tip Distance

**Figure 1:**
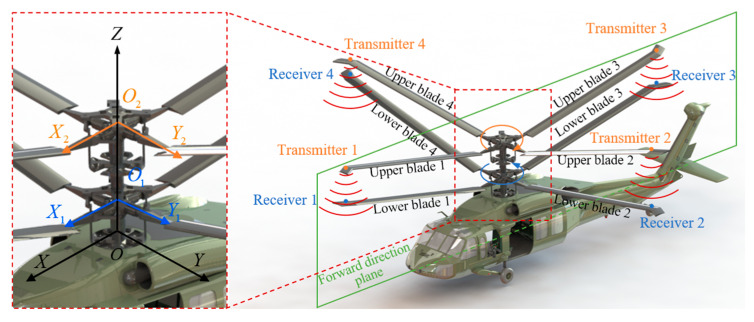
A schematic diagram of blade-tip distance measurement scheme based on ultrasonic ranging.

As shown in Figure 1, using an eight-blade coaxial helicopter as an example, the upper and lower rotors rotate at the same speed but in opposite directions. We define the helicopter’s fuselage coordinate system as *O-XYZ*, with *OZ* as the coaxial axial direction, *OX* as the forward direction plane of the helicopter, and *OY* as perpendicular to the forward direction plane of the helicopter. *O*_1_*-X*_1_*Y*_1_*Z* and *O*_2_*-X*_2_*Y*_2_*Z* represent the lower and upper rotor coordinate systems. The distance between the helicopter fuselage’s top plane and the lower rotor blade plane is defined as *OO*_1_ = *l*, and the distance between the lower and upper rotor blade planes is defined as *O*_1_*O*_2_ = *h*. There are eight intersections between the upper and lower blades during one revolution. Figure 1 represents a situation when each upper blade aligns with one lower blade. In the proposed measurement method, four ultrasonic transducers are located at the upper blade tip as transmitters, and another four transducers are secured at the lower blade tip as receivers. The positions of the transmitters and receivers are all the same distance away from the axis of the rotor system. Here, the ultrasonic ranging method adopts the pitch-catch configuration since the intersection time of the blades is short. The control module and the signal processing module are installed in the helicopter fuselage, and the signal transmission between the fuselage and the rotors is realized through the slip ring. A measurement is required at each blade intersection to measure the distance between each transmitter and receiver pair.

**Figure 2:**
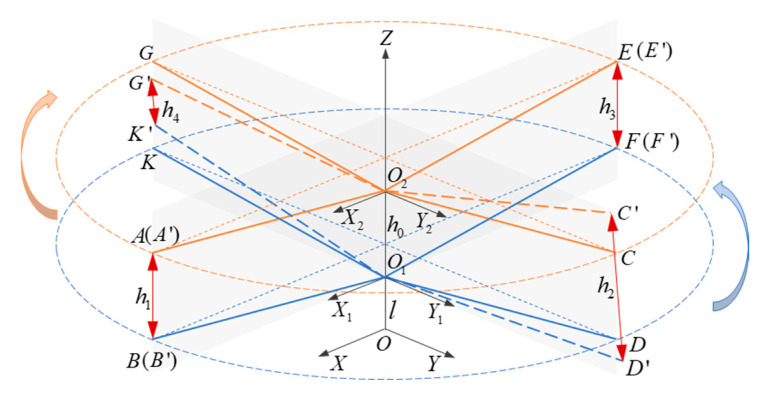
A 3D coordinate model for blade-tip distance measurement.

Figure 2 shows a three-dimensional (3D) coordinate model for the distance measurement of coaxial rotor blades. Points *A* to *G* and *K* are the theoretical positions of the blade tip at a certain time of intersection. However, the actual motion of the blade tip can deviate from the theoretical trajectory; thus, Points $A^{\prime }$ to $G^{\prime }$ and $K^{\prime }$ represent the possible positions during the motion. The distances to be measured are the blade-tip distances $h_{1}$, $h_{2}$, $h_{3}$, and $h_{4}$ at each intersection moment, and $h_{1}=A^{\prime }B^{\prime }$, $h_{2}=C^{\prime }D^{\prime }$, $h_{3}=E^{\prime }F^{\prime }$, and $h_{4}=G^{\prime }K^{\prime }$.

**Figure 3:**
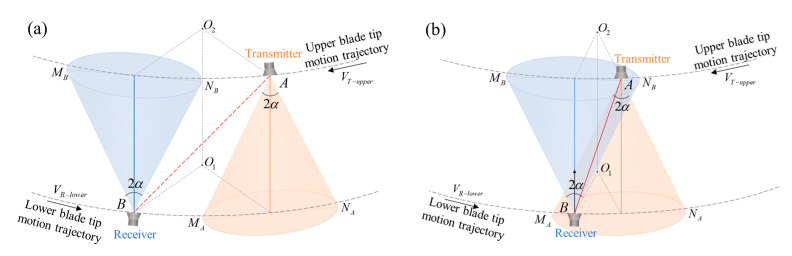
The positions of transmitter and receiver (**a**) out of the measurable range and (**b**) in the measurable range.

The measurable range of the intersection process is defined as the 3D space in which the receiver can receive the ultrasonic sequence transmitted by the transmitter. In general, the ranging system takes the corresponding space within the beam spread angle of the transducer as the measurable range. The measurable range mainly depends on the directivity of the transducer [14]. The parameter beam spread angle 2α can be used to represent the directivity of the ultrasound transducer. The beam spread angle measures the width of the beam, from side to side in degrees, of the main lobe where the ultrasonic energy intensity drops to −3 dB. In this paper, the directivity of the transducer is represented by the measurable range where effective signal acquisition and measurement can be completed. The cones in Figure 3 illustrate the simplified effective region in which the transducers transmit and receive ultrasonic waves. Effective measurement cannot be completed when the effective regions of the transmitter and receiver do not overlap, as shown in Figure 3a. When the blade tips are in the state shown in Figure 3b, the distance measurement can be completed as long as the two transducers stay within each other’s effective regions. Since the blade tip speed is relatively high during helicopter flight, turbulence can impact ultrasonic measurements. Specifically, turbulence affects the overlapping duration of the transmitter and receiver being within the measurable range, as well as the propagation of the ultrasonic measurement signal. Therefore, it is necessary to improve the subsequent requirements for measurement signals to ensure accurate identification of the measurement signal even under turbulent conditions.

**Figure 4:**
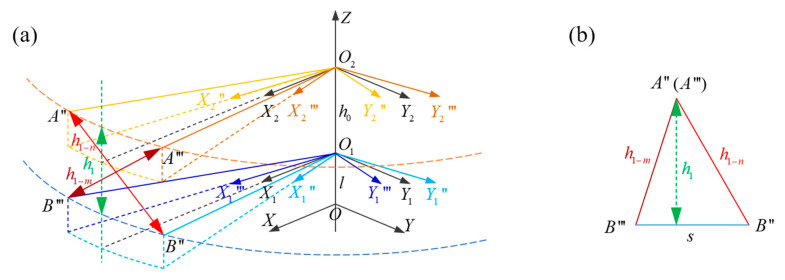
A coordinate system for blade-tip distance measurement at the intersection (**a**) Three-dimensional coordinate system and (**b**) the transformation to a two-dimensional triangle.

In the actual measurement, the measured distance (the red solid line in Figure 3b) may not be the vertical distance (the blue solid line in Figure 3b). Therefore, to calculate the vertical blade-tip distance ($h_{1}$), as is shown in Figure 4, at least two valid measurements need to be completed during each intersection. Points A″, B″, A‴, and B‴ are the positions of the blade tips when two measurements are completed, and the measured distances are $h_{1-n}$ and $h_{1-m}$, respectively. The measured distances are calculated by multiplying the speed of sound (*c*) and the measured time of flight (*Tof*) between the transmitted signal and the received signal. *s* is the sum of the distances moved by the upper blade tip and the lower blade tip, and s=A″A‴+B″B‴. Then,

(1) $$h_{1}=\frac{2\sqrt{p(p-h_{1-m})(p-h_{1-n})(p-s)}}{s}$$

where $p=(h_{1-n}+h_{1-n}+s)/2$.

To obtain the vertical blade-tip distance, multiple distances need to be measured in a very short intersection time, which puts forward high requirements in terms of the measurement rates for ultrasonic ranging. In the case of more than two distance values being obtained, the shortest two should be selected for calculation.

**Figure 5:**
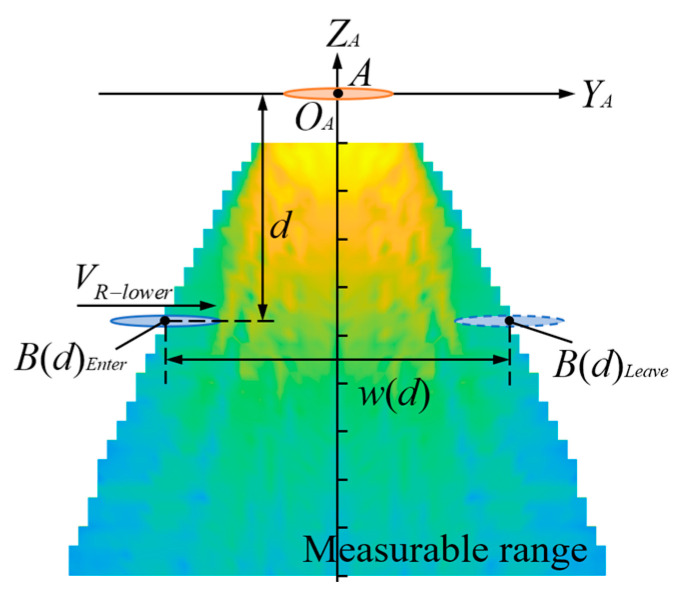
A schematic diagram of the measurement rate requirement at the intersection.

The requirement for the measurement rate during blade intersection depends on the duration of the transmitter and receiver being in the measurable range, which is shown in Figure 3b. With blade tip *A* as a reference, the coordinate system *O_A_*-*X_A_Z_A_* is established as shown in Figure 5. Blade tip *A* is relatively stationary, blade tip *B* moves in the positive direction along the *Y_A_* axis, and blade-tip distance measurement can be completed when blade tip *B* moves within the measurable range. The measurable range in Figure 5 is the measured range of the *O_A_*-*X_A_Z_A_* plane in the blade-tip distance *d* range of 100–1000 mm. The coordinates of blade tip *B* when it enters the measurable range and leaves the measurable range are $B{\left(d\right)}_{\mathrm{Enter}}(y_{Enter},\text{-}d)$ and $B{\left(d\right)}_{\mathrm{Leave}}(y_{Leave},\text{-}d)$, respectively. Then, the distance moved within the measurable range is $w(d)\;=\;y_{Leave}-y_{Enter}$. The speed of the blade tip *B* is $V_{R\text{-}\mathrm{Lover}}$, the duration within the measurable range is $T_{B}{\left(d\right)}_{\min}=w(d)/V_{R\text{-}\mathrm{Lover}}$, and the minimum time is $T_{B}{\left(d\right)}_{\min}=w(100)/V_{R\text{-}\mathrm{Lover}}$. Therefore, the requirement for the measurement rate is greater than $2/T_{B}{\left(d\right)}_{\min}$. The maximum speed of helicopter blade tips is generally less than 200 m per second, as it is limited by the speed of sound. The transmitter and receiver are installed at a distance of 0.85 radii from the axis, resulting in a relative blade tip speed $V_{R\text{-}\mathrm{Lover}}=340\;m/s$. Since the shortest measured distance is 100 mm, and w(100) is 320 mm according to the parameters of the transducer used, the required measurement rate needs to be greater than 2125 Hz.

### 2.2. Method of Improving Measurement Rate

The measurement rate is the reciprocal of the time required for a single measurement. When a pair of transducers in the pitch-catch mode is used in the ranging system, the transmitter would usually be set up in a way so that it will not fire the next pulse until the receiver receives the signal.

The maximum measurement rate of the pitch-catch method is as follows:

(2) $$\mathrm{Maximum}\;\mathrm{measurement}\;\mathrm{rate}=\frac{1}{\mathrm{Tof}}=\frac{c}{h}$$

where *h* is the measured distance.

The measurement rate decreases inversely with increases in the distance and is limited by the ultrasonic propagation speed. For a 1.0 m measurement distance, the measurement rate is 340 Hz, given that the speed of sound in air is 340 m/s at 20 °C.

**Figure 6:**
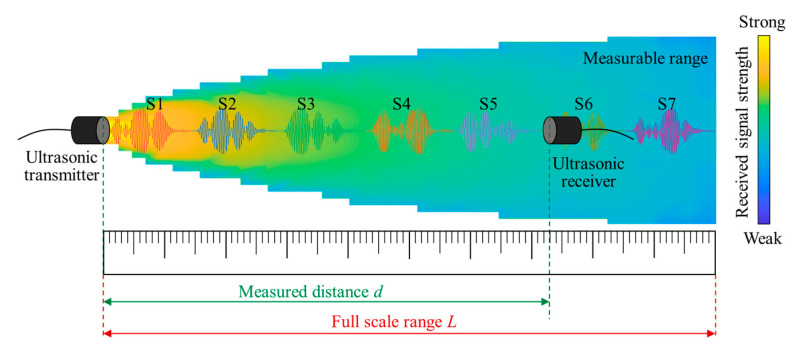
A schematic diagram of coded ultrasonic ranging.

According to the measurement rate requirement of the coaxial helicopter, it needs to be greater than 2125 Hz if two distance measurements need to be completed. Therefore, the measurement rate of the traditional pitch-catch method cannot meet the requirement for the measurement of blade-tip distance. To this end, we proposed the coded ultrasonic ranging method to improve the measurement rate. Instead of transmitting one measurement signal and waiting for the signal to fully propagate to the receiver, the transmitters will transmit a series of coded ultrasonic sequences with varying patterns. A signal processing module will be set up at the receiving end and complete the decoding of the received ultrasonic sequences. The sequence identification and associated transmitting time will be decoded to realize more measurements during the short period of blade intersection. Figure 6 illustrates the proposed coded ultrasonic ranging method. The ultrasonic transmitter transmits a series of coded ultrasonic signals of different characteristics in the measurable range.

Consider the single measurement period of the ultrasonic receiver within the measurable range as *T*, the number of coded signals being transmitted as η, and the maximum distance to be measured as *L*. Then, the number of codes η needs to satisfy the following formula:

(3) η≥L/(T⋅c)

Since the single measurement time needs to be less than 0.49 ms within a distance range of 1 m, according to Equation (3), when *T* = 0.49 ms, *L* = 1 m, and *c* = 340 m/s, then the number of coded signals η needs to be greater than six. In order to shorten the single measurement cycle, the signal length is also required, and the transmitted signal duration and received signal duration of each ultrasonic sequence should be less than *T*/1.5 ≈ 0.32 ms.

**Figure 7:**
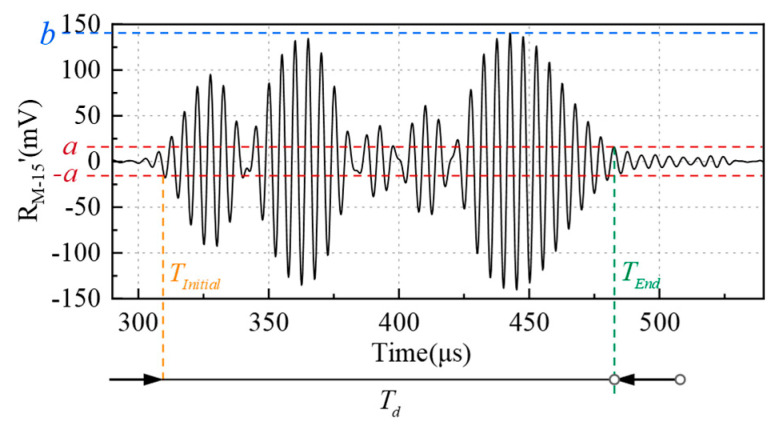
Definition of received signal duration $T_{d}$.

In addition, a single measurement *T* is determined by the maximum duration of the received signal $T_{d}$. To prevent aliasing between the received signals of different sequences, $T\geq 1.5T_{d}$ is set. $T_{d}$ is determined by the amplitude threshold method, which is a method to judge the effectiveness part of a signal according its voltage amplitude. As shown in Figure 7, two amplitude thresholds, *a* and *b*, are defined, where *b* is the maximum peak value of the received signal and *a* = *b*/10, since any amplitude less than *b*/10 is greatly affected by noise. The first sampling point whose amplitude is greater than the threshold value *a*, or less than −*a*, is taken as the initial point of the effective signal. The first sampling point whose amplitude is smaller than the threshold value and the subsequent sampling points that are less than the threshold value are taken as the end point of the effective signal. The initial point and the end point are defined as $T_{\mathrm{Initial}}$ and $T_{\mathrm{End}}$. The duration of a coded signal can then be calculated by $T_{\mathrm{End}}$ − $T_{\mathrm{Initial}}$.

Since the maximum measurement rate is limited by the maximum duration of the received signals $T_{d}$ and the number of codes η, the maximum measurement rate $v_{\max}$ based on a set of sequences can be defined as:

(4) $$v_{\max}=\min(1/1.5T_{d},\eta \cdot c/L)$$

## 3. Coded Ultrasonic Ranging

### 3.1. Measurement System

**Figure 8:**
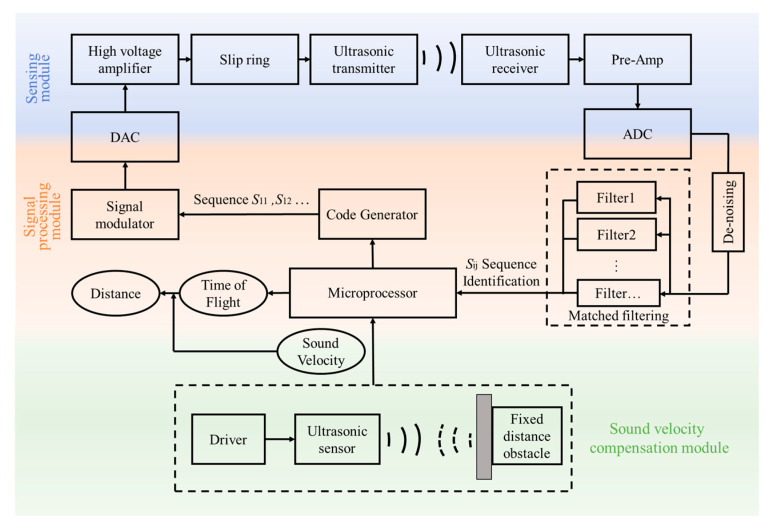
A block diagram of the proposed distance measurement method using coded ultrasonic ranging.

The proposed measurement system consists of a sensing module, a signal processing module, and a sound velocity compensation module. Figure 8 shows a block diagram of a complete distance measurement. The sensing module amplifies the modulated excitation signal and transmits it through the slip ring to the ultrasonic transmitter. After the coded ultrasonic wave is propagated in the air, the ultrasonic receiver receives the signal and amplifies it through the preamplifier. The signal processing module carries out the coding of the transmitted signal, the de-noising and decoding by the matched filter of the received signal, and the distance calculation by using a microprocessor. The sound velocity compensation module measures the known fixed distance using a calibrated ultrasonic sensor and obtains the real-time sound velocity. This compensation method can compensate for the errors caused by various environmental factors. The calculated distance is the distance *d* between the ultrasonic transmitter and the ultrasonic receiver. This paper presents our intermediate research and results on the proposed signal processing module, with an emphasis on the coding and decoding of the ultrasonic signal.

### 3.2. Coding Method of Excitation Signals

#### 3.2.1. Selection of Excitation Signals

For traditional ultrasonic ranging, the excitation signal is a sinusoidal pulse with a fixed period number, and the received signal can be generally modelled as a damped sinusoid. The received signal reflects the characteristics of the excitation signal to a certain extent. In order to make the received signals have different characteristics, it is necessary to encode the excitation signals.

Binary pseudorandom noise (PRN) sequences are a set of special ascertained vectors with outstanding autocorrelation and cross-correlation properties, i.e., orthogonality, such as Barker codes [17], Golay codes [18,19], M-sequences [20,21], Gold codes [22], and Kasami codes [23,24] to name a few. They add features of the binary sequence to the excitation signal and then manifest in the received signal. Among the above-mentioned coded signals, the length of each Barker code is inconsistent, which will cause the receiving signal duration to be different; Golay codes have good autocorrelation characteristics and can eliminate sidelobe; however, the signals need to be transmitted twice which is not suitable for high-speed and dynamic measurement; the length of Kasami codes is longer than that of M-sequence and Gold codes, making them less favourable when the signal length and measurement rate matter. Therefore, M-sequence and Gold codes are more suitable for our application of high-speed measurement.

For the efficient transmission of PRN sequences via an ultrasonic transducer, the signal must be generated using a specific modulation scheme. Common modulation methods are binary amplitude shift keying (BASK) [25], binary frequency shift keying (BFSK) [26], and binary phase shift keying (BPSK) [27]. When the excitation signal adopts a rising edge and a falling edge, the received signal has a different starting vibration direction, so the BPSK of the excitation signal can be used to change the vibration form of the received signal.

#### 3.2.2. M-Sequences and Gold Codes

After considering the signal length and the number of codes, M-sequences and Gold codes are chosen. An M-sequence, or maximum-length sequence, is a type of pseudorandom binary sequence with the property of having the maximum possible length for a given register size. These sequences are generated using linear feedback shift registers (LFSRs) and exhibit a maximum period of $2^{k}-1$, where *k* is the number of shift register stages. M-sequences are characterized by their maximal length, pseudo-randomness, and unique autocorrelation properties. A Gold code is a composite sequence of M-sequences, which is composed of two M-sequence pairs with the same code appearance and code clock rate through mod 2 operation. Gold codes can generate more sequences, and their cross-correlation property is better than that of M-sequences. In this article, 7-bit M-sequences, 15-bit M-sequences, and 7-bit Gold codes are used for the coding ranging system because the number of sequences is 7, 15, and 9, respectively, which meets the requirements of >6.

The coded sequences are defined as $s_{Mi\text{-}j}(m)$ and $s_{\mathrm{Gold}\;i\text{-}j}(m)$, where *i* represents the number of sequence groups and *j* represents the position of the sequence within its respective group. The two groups of 7-bit M-sequences are defined as M1 and M2, the two groups of 15-bit M-sequences are defined as M3 and M4, and the seven groups of Gold codes are defined as Gold1, Gold2, …, and Gold7 respectively. The 7-bit M-sequences and 7-bit Gold codes consist of 7 code values, the 15-bit M-sequences consist of 15 code values, and *m* represents the specific position of the code value. Take M1 as an example, when *j* = 1, $s_{M1\text{-}1}(m)=$ [1, 0, 1, 1, 1, 0, 0] The other sequences of each set of M-sequences are obtained by cyclic shifting of the first sequence. For Gold codes, the first group is calculated by these two M-sequence pairs, [1, 0 ,1 ,1 ,1 ,0 ,0] and [1, 0, 1, 0, 0, 1, 1], and the other groups are obtained by the first group of cyclic shifts. The specific code values are shown in Table 1, Table 2 and Table 3.

#### 3.2.3. Coded Excitation Signals

In this research, the BPSK method is adopted, and the carrier wave is a sine wave with *q* cycles at the transducer’s resonance frequency.

The coded excitation signal of the M-sequences and Gold codes is defined as $E_{Mi\text{-}j}(n)$ and $E_{\mathrm{Gold}\;i\text{-}j}(n)$, respectively, and

(5) $$E_{Mi\text{-}j}(n)=U\cdot \sum_{M=1}^{p}\sin\{2\pi f(n/f_{s})+[s_{Mi\text{-}j}(m)+1]\cdot \pi \}*\delta [n\text{-}\frac{f_{s}}{f}\cdot q(m-1)]$$

(6) $$E_{\mathrm{Gold}\;i\text{-}j}(n)=U\cdot \sum_{M=1}^{p}\sin\{2\pi f(n/f_{s})+[s_{\mathrm{Gold}\;i\text{-}j}(m)+1]\cdot \pi \}*\delta [n\text{-}\frac{f_{s}}{f}\cdot q(m-1)]$$

where *U* is the amplitude of the excitation voltage, $s_{Mi\text{-}j}(m)$ and $s_{\mathrm{Gold}\;i\text{-}j}(m)$ are the coded sequences, $n\;=\;0,1,2,\ldots ,(q\cdot f_{s}/f\;-\;1)$ and *n_max_* + 1 = $q\cdot f_{s}/f$ are the number of sample points of the excitation signal corresponding to each code value, $f_{s}$ is the signal acquisition frequency, *p* is the number of bits of the sequence, *f* is the center frequency of the transducer, δ(n) is the unit impulse function, and * refers to the convolution operation.

**Figure 9:**
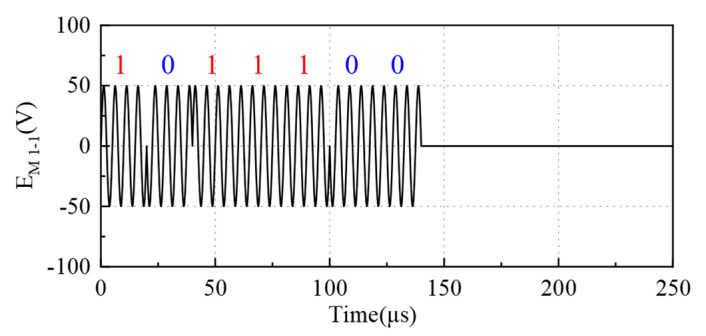
An excitation signal modulated by an M-sequence $s_{M1\text{-}1}$.

Higher transducer center frequencies lead to faster energy attenuation during propagation, but also shorter single-cycle vibration times and thus a higher resolution. Considering that the measured distance range is up to 1 m, the transducer with a center frequency of 200 kHz is selected. The received signal is longer than the excitation signal due to the transducer’s residual vibration. To meet the requirement that the period is less than 0.32 ms, the carrier cycles choose 4 and 2, and so the excitation signal lengths are 0.14 ms and 0.15 ms. Under this condition, the received signal at 1 m can also ensure better quality. Taking $E_{M1\text{-}1}(n)$ as an example, the coded excitation signal is shown in Figure 9.

### 3.3. Decoding Method of Received Signals

#### 3.3.1. De-Noising Method

The ultrasonic transmitter emits an ultrasonic wave in response to a coded excitation signal. The receiver converts the returning signal to an electrical form and digitizes it with an AD converter at 5 MHz. Filtering is then used to remove noise before decoding. In this paper, a bandpass filter and wavelet filter are investigated for the coded signal that is used and their filtering effects are evaluated by the resulting degree of signal restoration and received signal correlation. The smaller the degree of signal distortion, the less the influence on the accuracy of the subsequent calculation of *Tof*. The received signal correlation refers to the degree of correlation between the filtered received signals and the same code under different conditions. The higher the correlation, the more conducive the signal is to subsequent decoding processing.

A bandpass filter is a filter that passes through frequency components in a certain frequency range but attenuates frequency components in other ranges to very low levels. The passband cut-off frequencies are *f_p_*_1_ = 150 kHz and *f_p_*_2_ = 250 kHz, the stopband cut-off frequencies are *f_s_*_1_ = 50 kHz and *f_s_*_2_ = 350 kHz, the maximum passband attenuation is 2 dB, the minimum stopband attenuation is 30 dB, and the order of the filter is 5. The wavelet filter [28] is a powerful tool for digital signal processing which decomposes a signal in both the frequency and time domains. The factors that affect the effect of noise reduction include the wavelet basis, decomposition layer number, threshold value, and threshold function. Here, the db4 wavelet is selected, for which the wavelet function is Daubechies and the order of vanishing moments is 4, making it suitable for the decomposition of one-dimensional signals. The number of decomposition layers is four, which is determined by the sampling rate of 5 MHz. The threshold is selected by the ‘*VisuShrink*’ method and $\lambda =\sigma \sqrt{2\ln N}$, where σ is the noise standard deviation and *N* is the signal length, and the threshold function is selected by the soft threshold function due to the better overall continuity of the signal.

**Figure 10:**
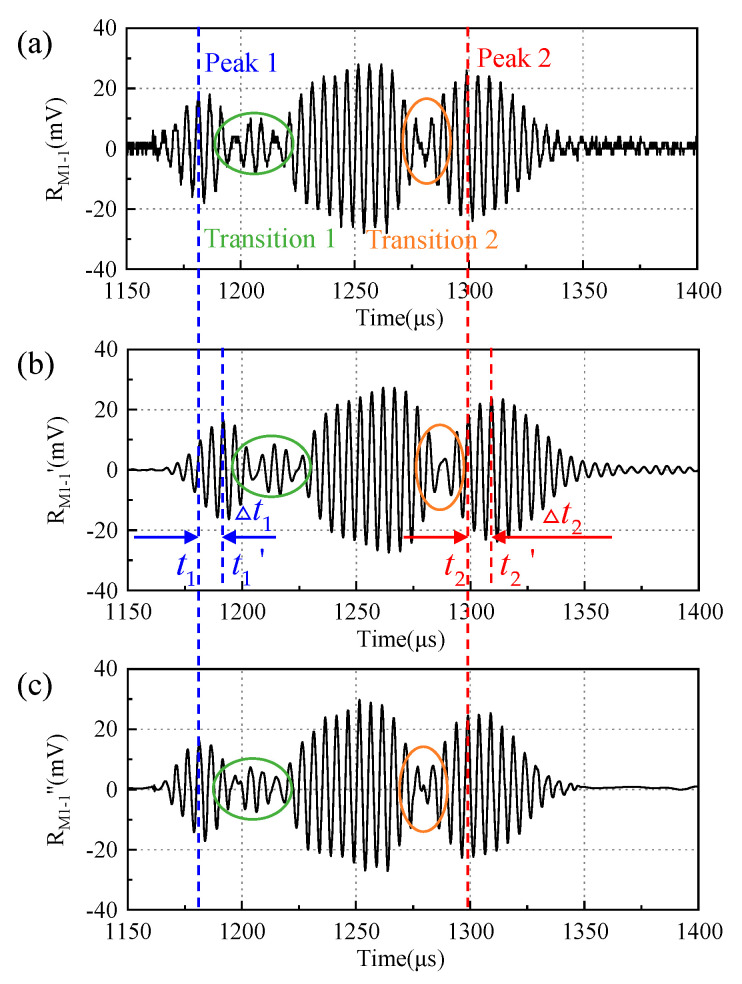
The received signals of an M-sequence: (**a**) unprocessed received signal, (**b**) received signal after bandpass filtering, and (**c**) received signal after wavelet filtering.

Taking the M-sequence as an example, $R_{Mi\text{-}j}(n)$ is the unprocessed received signal of a coded sequence and $R_{Mi\text{-}j^{\prime }}(n)$ and $R_{Mi\text{-}j}\prime \prime (n)$ are the received signal after bandpass filtering and wavelet filtering, respectively. The filtering results are presented in Figure 10.

Comparing the two methods based on the degree of signal restoration, the wavelet filter proves superior. It provides more accurate restoration, particularly at the transition points (circled in Figure 10), without the time delay observed (for example, at the peak-1 and peak-2 positions in Figure 10).

The correlation performance is determined by ρ(d).

(7) $$\rho (d)=\left(\sum_{j=1}^{\eta }\rho_{Mi\text{-}j}(d)\right)/\eta$$

where *d* is the reference distance between the transmitter and the receiver, η is the number of sequences in each group, $\rho_{Mi\text{-}j}(d)$ is the correlation coefficient between the received signal at *d* and the received signal at *d*_min_ = 100 mm of the same coding, and the calculation formula is as follows:

(8) $$\rho_{Mi\text{-}j}(d)=\frac{\sum \left(R_{Mi\text{-}j}(d_{\min})-\bar{R_{Mi\text{-}j}(d_{\min})}\right)\left(R_{Mi\text{-}j}(d)-\bar{R_{Mi\text{-}j}(d)}\right)}{\sqrt{\sum {\left(R_{Mi\text{-}j}(d_{\min})-\bar{R_{Mi\text{-}j}(d_{\min})}\right)}^{2}}\sqrt{\sum {\left(R_{Mi\text{-}j}(d)-\bar{R_{Mi\text{-}j}(d)}\right)}^{2}}}$$

**Figure 11:**
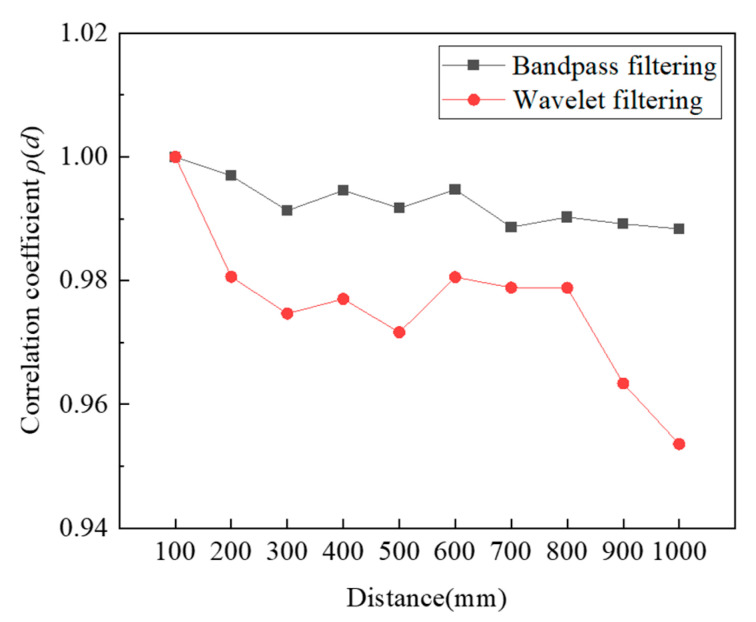
The correlation coefficient of the received signal after filtering.

The calculated correlation coefficients of the received signals after bandpass filtering and wavelet filtering, respectively, are shown in Figure 11. Comparing the two methods based on the received signal correlation at various distances, the bandpass filtering shows a higher correlation coefficient than the wavelet filtering.

Due to the consistency of the delay in the time domain, it can be compensated for by calculating the *Tof*. In addition, the distortion at the transition position has little effect on the calculation of the *Tof* as long as the signal’s starting point is clear. Therefore, the received signal after the bandpass filtering is chosen for subsequent decoding.

#### 3.3.2. Decoding Method

The decoding process determines the excitation signal by identifying the corresponding coded sequence within the received signal. This is achieved using a matched filter. The filtered received signal is cross-correlated with each reference signal, generating a set of cross-correlation values. The coded sequence associated with the highest cross-correlation value is the decoded result. $X_{Mi\text{-}j}(n)$ is the reference signal corresponding to $s_{Mi\text{-}j}$. The reference signal is the filtered signal when the excitation voltage is 200 Vpp and the distance between the transmitter and the receiver is 100 mm. The length of the reference signal is 0.25 ms, the same as received signal $R^{\prime }(n)$.

(9) $$\rho_{Mi\text{-}j}=\left(\sum \left(R^{\prime }-\bar{R^{\prime }})(X_{Mi\text{-}j}-\bar{X_{Mi\text{-}j}}\right)\right)/\left(\sqrt{\sum {\left(R^{\prime }-\bar{R^{\prime }}\right)}^{2}}\cdot \sqrt{\sum {\left(X_{Mi\text{-}j}-\bar{X_{Mi\text{-}j}}\right)}^{2}}\right)$$

For a given set of sequences, *i* is a fixed value and the $\rho_{Mi\text{-}j}$ values corresponding to different *j* values are calculated. The decoding result is *j*, corresponding to the maximum value of $\rho_{Mi\text{-}j}$. That is, the code value $s_{Mi\text{-}j}$ of the excitation signal corresponding to the received signal $R^{\prime }(n)$ is obtained.

#### 3.3.3. Coding Performance Evaluation Method

A coding group is deemed effective when the received signals for each code within the group can be correctly decoded at different distances. The performance of these effective coding groups is then evaluated and compared. Two key criteria are used for this assessment: the distinguishability of the received signals and their energy. The objective function is as follows.

(10) $${\mathrm{Ob}}_{1}≜\min((\rho_{Mi\text{-}1\max}-\rho_{Mi\text{-}1\mathrm{submax}}),(\rho_{Mi\text{-}2\max}-\rho_{Mi\text{-}2\mathrm{submax}}),\ldots (\rho_{Mi\text{-}\eta \max}-\rho_{Mi\text{-}\eta \;\mathrm{submax}}))=\max$$

(11) $${\mathrm{Obj}}_{2}≜\min(P_{Mi\text{-}1},P_{Mi\text{-}2},\ldots ,P_{Mi\text{-}\eta })=\max$$

where $\rho_{Mi\text{-}j\max}(j=1,2,\ldots ,\eta )$ is the correlation coefficient of the received signal and its corresponding reference signal, $\rho_{Mi\text{-}j\mathrm{submax}}(j=1,2,\ldots ,\eta )$ is the maximum value among the correlation coefficients calculated from the received signal and other reference signals, and $P_{Mi\text{-}j}(j=1,2,\ldots ,\eta )$ is the energy of the received signals corresponding to the *j*th sequence. The first index guarantees the optimal correlation properties, while the second one maximizes the energy of the received signals and makes the signal-to-noise ratio higher.

## 4. Experiment Results and Discussion

### 4.1. Experiment Set-Up

**Figure 12:**
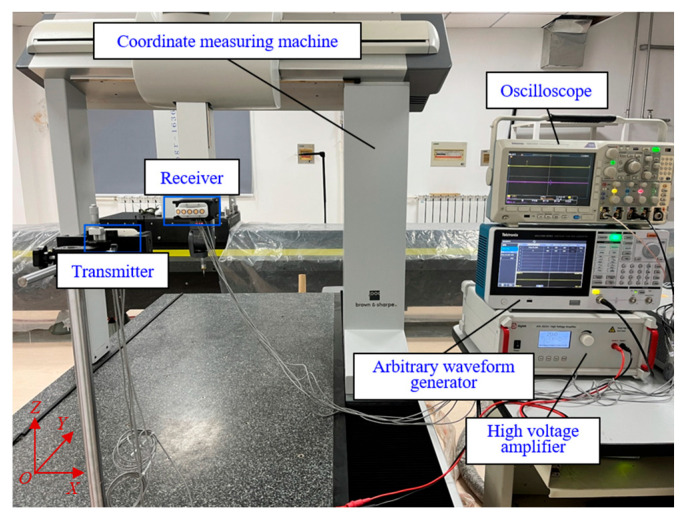
The experiment platform of the coded ultrasonic ranging system.

An ultrasonic coding ranging experimental setup which was used to validate the proposed method is shown in Figure 12. The transmitter is fixed, and the receiver is installed at the probe of the coordinate measuring machine. The coded excitation signals generated by the arbitrary signal generator are applied to the transmitter through a high-voltage amplifier. The response of the receiver is collected through the oscilloscope and then processed by the computer. After considering the center frequency, directivity, ultrasonic propagation characteristics, and size of the ultrasonic transducer, DYA-200-01B ultrasonic transducers (Hangzhou Umbrella Automation Technology Co., Ltd., Hangzhou, China), which have a center frequency of 200 kHz and a beam angle of 14.5°, are used as the transmitter and the receiver. In Figure 12, it is shown that only the middle transducers in both transmitter and receiver housings are activated in this experiment. The test platform is a coordinate measuring machine (Global Classic SR 07.10.07, Hexagon Measurement Technology Ltd., Qingdao, China). The amplitude of the output signal from an arbitrary signal generator (AFG31021, Tektronix, Beverly Hills, OR, USA) is set to 5 Vpp. A high-voltage amplifier with a bandwidth of 1 MHz (ATA-2000, Aigtek, Xi’an, China) is used to amplify the ultrasound signal due to the attenuation of sound in the air. The output voltage of the high-voltage amplifier is 50 Vpp after the magnification factor is set to 10 times. The sampling rate of the oscilloscope is set to 5 MS/s. In addition, since the experiment in this paper is intended to evaluate the performance of different coding methods, it is necessary to fix the transducer on the experimental platform to accurately collect the received signals at each measuring point. Thus, it is not necessary to move the transducers while collecting the received signals in this experiment.

Firstly, the apertures of the transmitter and the receiver are carefully aligned at a distance of 0 between the transmitter and the receiver, and then the coordinate measuring machine probe is moved in the positive direction of the *Y* axis. The moving distance of the probe is taken as the reference distance. The measuring distance range is 100–1000 mm, and a measuring point is selected at every 100 mm increment.

### 4.2. Results of Coding and Decoding

**Figure 13:**
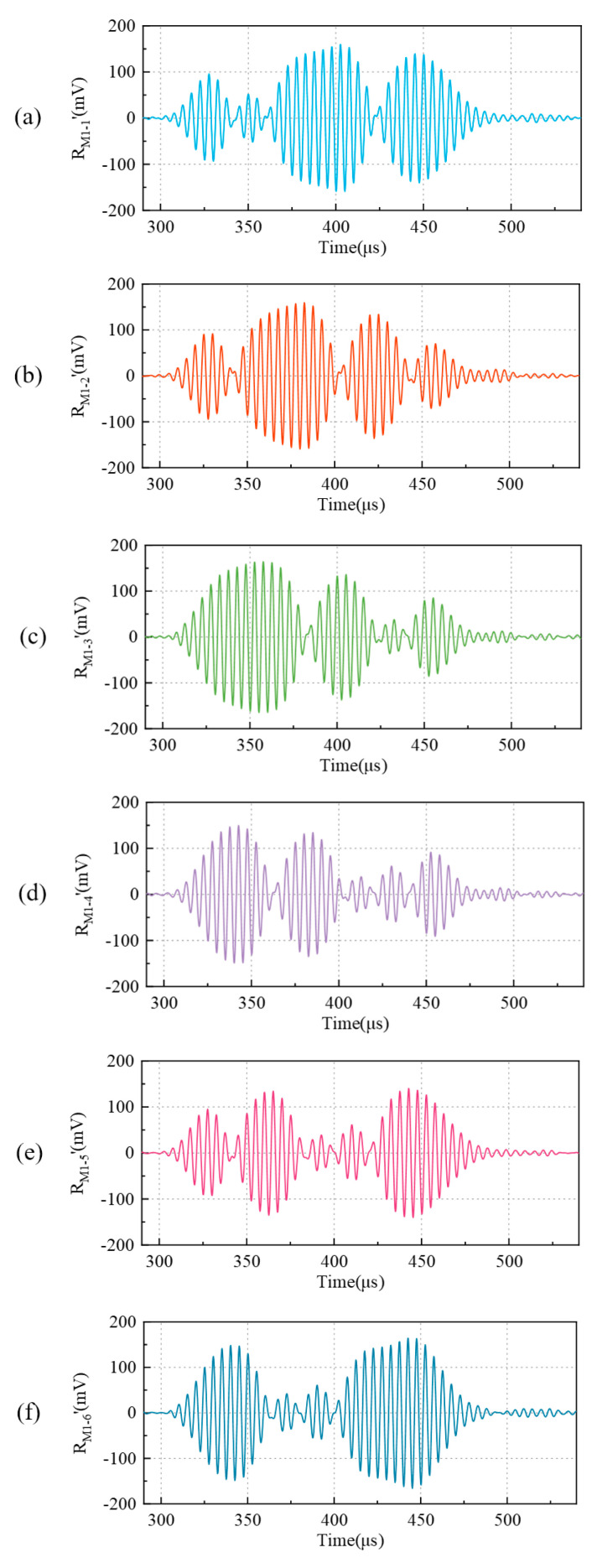

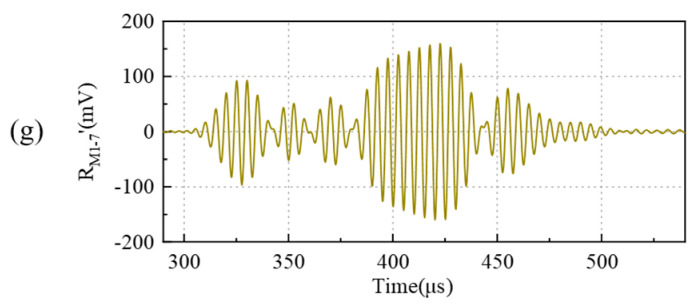
The results of received signals of sequences (**a**) M1-1, (**b**) M1-2, (**c**) M1-3, (**d**) M1-4, (**e**) M1-5, (**f**) M1-6, and (**g**) M1-7 at 100 mm.

Take M1 at 100 mm as an example, for which the received signals after filtering are shown in Figure 13. It can be seen that the response of the receiver is different for the different codes. The envelopes of the received signal waveform are different, which proves that their responses to different coded excitations are different. The consecutive identical codes in the coding are taken as the same segment signal response, and then the received signals for M1 are divided into four or five response segments. It can be seen that the received signals of seven sequences can correspond to their respective code values. Other M-sequences and Gold codes also represent different characteristics of the response. Therefore, the distinguishability and energy in Section 3.3.3 were evaluated.

The distinguishability of the coding is defined by parameter *u*, and *u* is defined as follows:

(12) $$u=\rho_{Mi\text{-}j\max}-\rho_{Mi\text{-}j\mathrm{submax}}$$

**Figure 14:**
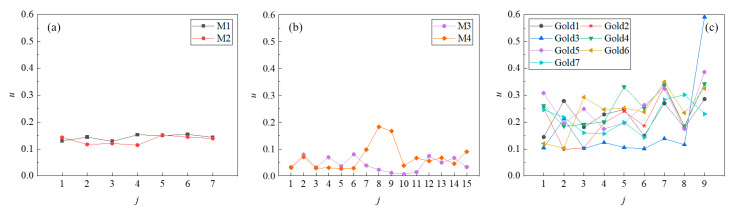
The distinguishability for different coding methods: (**a**) 7-bit M-sequences, (**b**) 15-bit M-sequences, and (**c**) 7-bit Gold codes.

A higher *u* means that the autocorrelation performance of the coded signal is better and that the cross-correlation performance is weaker, that is, the distinguishability is preferable. In this paper, the distinguishability is evaluated by the average of *u* for every 100 mm increment in the range of 100–1000 mm; the calculation results for different codes are shown in Figure 14. The minimum values of *u* for each sequence are shown in Table 4, since the minimum value determines the least distinguishable sequence of the group. It can be seen that the performance of the 7-bit M-sequences and 7-bit Gold codes is better than that of 15-bit M-sequences, since the *u*_min_ values of the 7-bit M-sequence and 7-bit Gold codes are around 0.1, and most of them are greater than 0.1, while the *u*_min_ of the 15-bit M sequence is less than 0.03. The *u*_min_ of the 15-bit M-sequence is close to 0, which leads to the risk of misjudgment and poor distinguishability. Therefore, the 7-bit M-sequences and the Gold codes are selected. Within the selected sequences, the two 7-bit M-sequences are equivalent because of their similar distinguishability, and Gold4 and Gold5 perform better than the other Gold codes and M-sequences since they have larger *u*_min_ values, 0.185 and 0.175, respectively. In addition, as shown in Figure 14, the *u* values of different code values in the same group of M-sequences are very close, and the maximum value is about 1.19–1.33 times the minimum value, while the *u* values of the Gold codes are very different, as the maximum value is between 1.85–5.85 times the minimum value. When the number of required code values decreases, the larger the ratio between the maximum and the minimum values, the better the distinguishability performance will be when some code values in the sequence are extracted.

**Figure 15:**
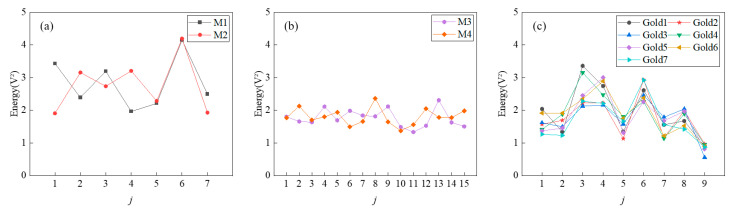
The energy of the received signals for different coding methods: (**a**) 7-bit M-sequences, (**b**) 15-bit M-sequences, and (**c**) 7-bit Gold codes.

As for the energy of the received signals, the average energy of the received signals at 1000 mm in 10 repeated experiments is evaluated, and the results of different codes are shown in Figure 15. The minimum values of energy for each sequence are shown in Table 4, since the minimum value determines the lowest-energy sequence of the group. The energy performance of the M-sequences is better than that of the Gold codes since the minimum values of energy of the 7-bit M-sequences are larger than 1.9 V^2^ and the minimum values of energy of the 15-bit M-sequences are larger than 1.3 V^2^, while the minimum values of energy of the 7-bit Gold codes are less than 1.0 V^2^. In addition, M1 is similar to M2, while M3 is similar to M4. As shown in Figure 15, the energy values of different code values in the same group of 7-bit M-sequences are similar, with the maximum value being from 2.11 to 2.20 times the minimum value. The energy values of the 15-bit M-sequences are more closely distributed compared to the 7-bit M-sequences, with the maximum value being from 1.72 to 1.73 times the minimum value. In contrast, the energy values of the Gold codes show relatively greater variation, with the maximum value being from 3.02 to 4.38 times the minimum value. When the number of required code values decreases, the larger the ratio between the maximum and the minimum values, the stronger the energy will be when some code values in the sequence are extracted. The higher the received signal energy, the less it is affected by noise, which is advantageous for signal processing in subsequent distance calculations.

According to the evaluation results of the sequences’ distinguishability and energy, 7-bit M-sequences and 7-bit Gold codes can be used as the code for blade-tip distance measurement. Then, the maximum duration of the received signal for different sets of sequences is calculated and shown in Table 5.

Combined with the maximum duration of the received signals $T_{d\text{-}\max}$ and the number of coded sequences η, according to Equation (4), the maximum measurement rates $v_{\max}$ of the 7-bit M-sequences and 7-bit Gold codes are shown in Table 6. All of these sequences can satisfy the measurement rate requirement (≥2125 Hz) of blade-tip distance measurement.

In summary, in terms of the number of codes, Gold codes have more codes, and thus a higher measurement rate (up to 3060 Hz) can be achieved. In terms of distinguishability, the Gold4 and Gold5 codes perform better, with *u*_min_ values of 0.185 and 0.175, respectively, which are larger than those of other codes. In terms of energy, the M1 and M2 codes perform better, with Energy_min_ values of 1.964 V^2^ and 1.909 V^2^, respectively, which are larger than those of other codes. Therefore, the code can be selected according to the actual needs.

## 5. Conclusions

This paper presents a method based on coded ultrasonic ranging for measuring the blade-tip distance of coaxial rotor helicopters. The feasibility of the method is proved theoretically and experimentally. Meanwhile, a method of improving the ultrasonic measurement rate is also proposed and the continuous measurement of ultrasonic ranging is realized. The influence of real-time flight environments, such as turbulence, on the measurement performance could be studied in the future.

The coding method of the excitation signal adopted herein is the BPSK mode of 7-bit M-sequences and Gold codes with a four sine waves carrier. The received signal is processed by wavelet filtering and bandpass filtering to denoise it, and the degree of signal restoration and the correlation of the received signal at different distances is taken as the evaluation index. Then, the bandpass filtering is chosen as the signal denoising method. The adopted method of decoding the received signal is cross-correlation operation through the matching filter, and the 7-bit M-sequences and Gold codes have been proven to be decodable. Therefore, they can be used in the coded ranging system to improve the measurement rate. A comparison between the results of this paper and those of other references is shown in Table 7.

The performance of different codes is evaluated based on the received signal distinguishability and energy. The Gold4 and Gold5 codes offer better distinguishability, while the M1 and M2 codes exhibit higher energy. The proposed coding and decoding methods meet the requirements for blade-tip distance measurement: (a) clear signal distinguishability, (b) a sufficient number of coded sequences, and (c) a short received signal duration.

## Figures and Tables

**Table 1:** Code values of 7-bit M-sequences.

7-bit M-Sequence	M1	M2
*j* = 1	[1, 0, 1, 1, 1, 0, 0]	[1, 0, 1, 0, 0, 1, 1]
*j* = 2	[0, 1, 1, 1, 0, 0, 1]	[0, 1, 0, 0, 1, 1, 1]
*j* = 3	[1, 1, 1, 0, 0, 1, 0]	[1, 0, 0, 1, 1, 1, 0]
*j* = 4	[1, 1, 0, 0, 1, 0, 1]	[0, 0, 1, 1 ,1 ,0 ,1]
*j* = 5	[1, 0, 0, 1, 0, 1, 1]	[0, 1, 1, 1, 0, 1, 0]
*j* = 6	[0, 0, 1, 0, 1, 1, 1]	[1, 1, 1, 0, 1, 0, 0]
*j* = 7	[0, 1, 0, 1, 1, 1, 0]	[1, 1, 0, 1, 0, 0, 1]

**Table 2:** Code values of 15-bit M-sequences.

15-bit M-Sequence	M3	M4
*j* = 1	[1, 0, 0, 1, 1, 0, 1, 0, 1, 1, 1, 1, 0, 0, 0]	[1, 0, 0, 1, 0, 0, 0, 1, 1, 1, 1, 0, 1, 0, 1]
*j* = 2	[0, 0, 1, 1, 0, 1, 0, 1, 1, 1, 1, 0, 0, 0, 1]	[0, 0, 1, 0, 0, 0, 1, 1, 1, 1, 0, 1, 0, 1, 1]
*j* = 3	[0, 1, 1, 0, 1, 0, 1, 1, 1, 1, 0, 0, 0, 1, 0]	[0, 1, 0, 0, 0, 1, 1, 1, 1, 0, 1, 0, 1, 1, 0]
…	…	…
*j* = 13	[0, 0, 0, 1, 0, 0, 1, 1, 0, 1, 0, 1, 1, 1 ,1]	[1, 0, 1, 1, 0, 0, 1, 0, 0, 0, 1, 1, 1, 1, 0]
*j* = 14	[0, 0, 1, 0, 0, 1, 1, 0, 1, 0, 1, 1, 1, 1, 0]	[0, 1, 1, 0, 0, 1, 0, 0, 0, 1, 1, 1, 1, 0, 1]
*j* = 15	[0, 1, 0, 0, 1, 1, 0, 1, 0, 1, 1, 1, 1, 0, 0]	[1, 1, 0, 0, 1, 0, 0 ,0, 1, 1, 1, 1, 0, 1, 0]

**Table 3:** Code values of 7-bit Gold codes.

7-bit Gold Code	Gold1	Gold2	…	Gold6	Gold7
*j* = 1	[1, 0, 1, 1, 1, 0, 0]	[0, 1, 1, 1, 0, 0, 1]	…	[0, 0, 1, 0, 1, 1, 1]	[0, 1, 0, 1, 1, 1, 0]
*j* = 2	[1, 0, 1, 0, 0, 1, 1]	[0, 1, 0, 0, 1, 1, 1]	…	[1, 1, 1, 0, 1, 0, 0]	[1, 1, 0, 1, 0, 0, 1]
*j* = 3	[0, 0, 0, 1, 1, 1, 1]	[0, 0, 1, 1, 1, 1, 0]	…	[1, 1, 0, 0, 0, 1, 1]	[1, 0, 0, 0, 1, 1, 1]
…	…	…	…	…	…
*j* = 7	[1, 1, 0, 0, 1, 1, 0]	[1, 0, 0, 1, 1, 0, 1]	…	[1, 0, 1, 1, 0, 0, 1]	[0, 1, 1, 0, 0, 1, 1]
*j* = 8	[0, 1, 0, 1, 0, 0, 0]	[1, 0, 1, 0, 0, 0, 0]	…	[0, 0, 0, 1, 0, 1, 0]	[0, 0, 1, 0, 1, 0, 0]
*j* = 9	[0, 1, 1, 0, 1, 0, 1]	[1, 1, 0, 1, 0, 1, 0]	…	[0, 1, 0, 1, 1, 0, 1]	[1, 0, 1, 1, 0, 1, 0]

**Table 4:** The minimum values of *u* and the energy of the received signals for different sequences.

Sequences	M1	M2	M3	M4	Gold1	Gold2	Gold3	Gold4	Gold5	Gold6	Gold7
$u_{\min}$	0.129	0.115	0.007	0.026	0.145	0.098	0.101	0.185	0.175	0.104	0.143
${\mathrm{Energy}}_{\min}/V^{2}$	1.964	1.909	1.335	1.375	0.897	0.972	0.561	0.945	0.815	0.897	0.873

**Table 5:** The maximum duration $T_{d\text{-}\max}$ of the received signals.

Sequences	M1	M2	Gold1	Gold2	Gold3	Gold4	Gold5	Gold6	Gold7
$T_{d\text{-}\max}/\mu s$	182.2	184.8	176.0	185.8	189.2	183.0	185.6	180.8	183.0

**Table 6:** The maximum measurement rate $v_{\max}$ for different sequences.

Sequences	*v*_max_
M1, M2	2380 Hz
Gold1, Gold2, Gold3, Gold4, Gold5, Gold6, Gold7	3060 Hz

**Table 7:** Comparison of measurement rates.

	Measurement Rate
Method presented in this paper	3060 Hz
[15]	143 Hz
[16]	100 Hz
[29]	455 Hz

## Data Availability

Data are contained within the article.
